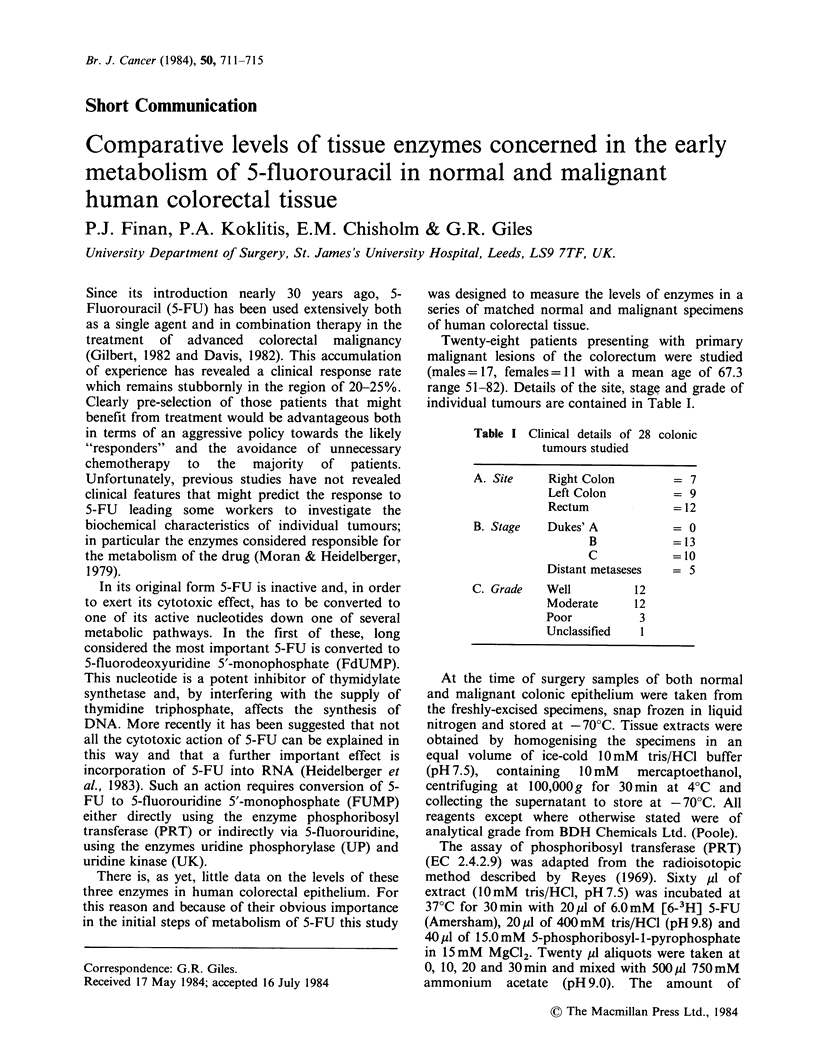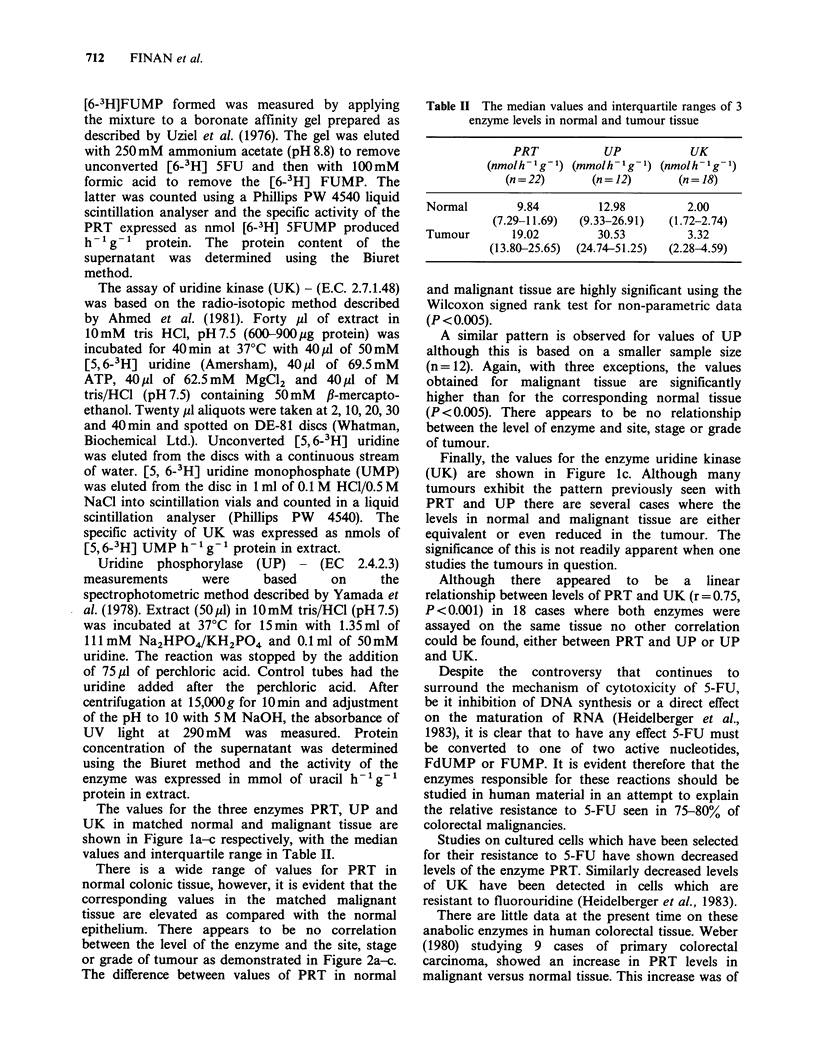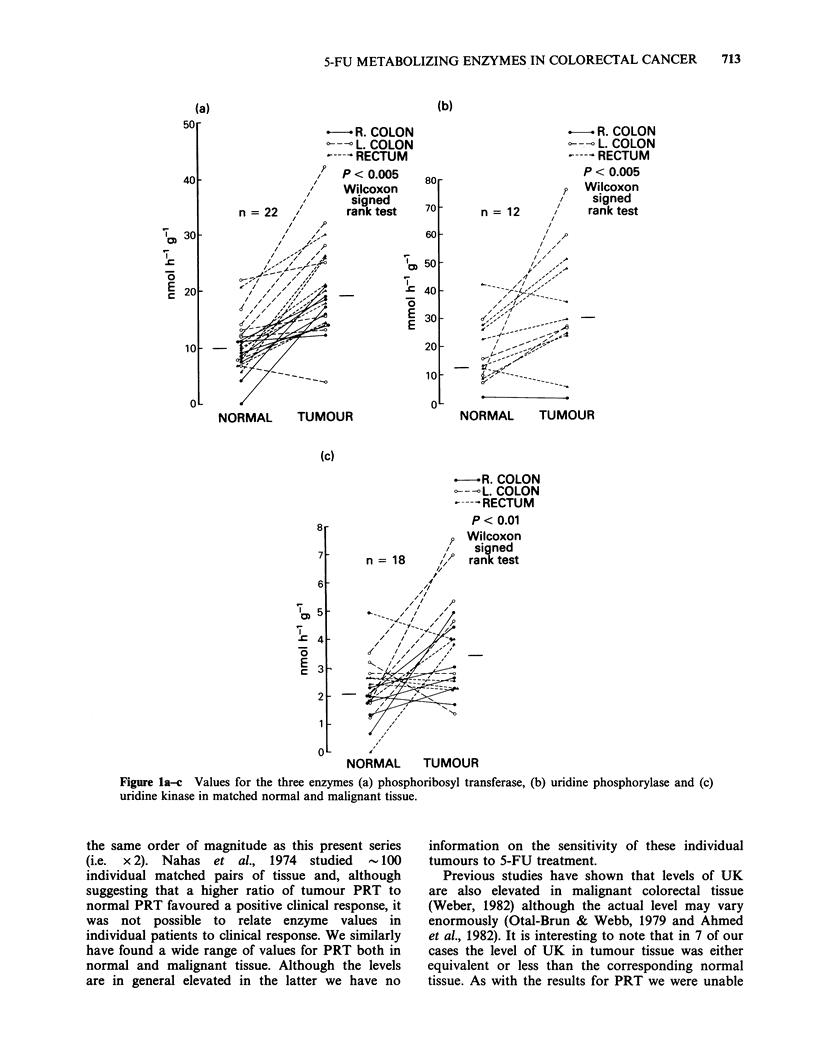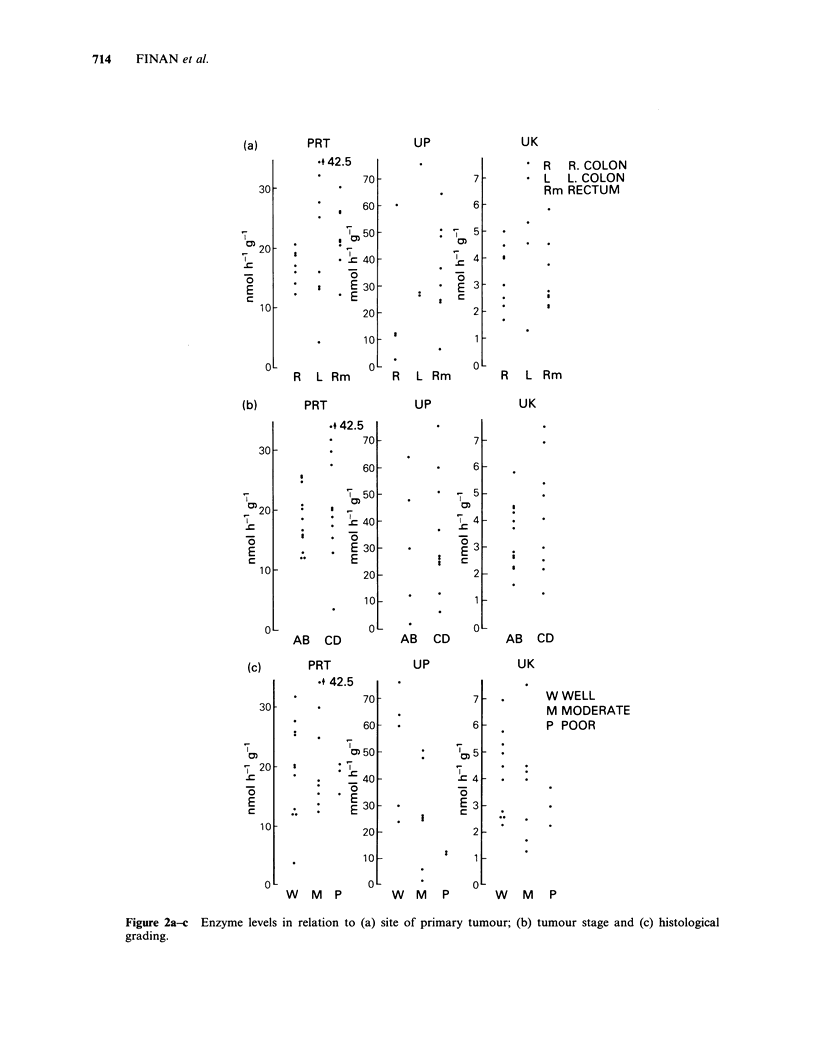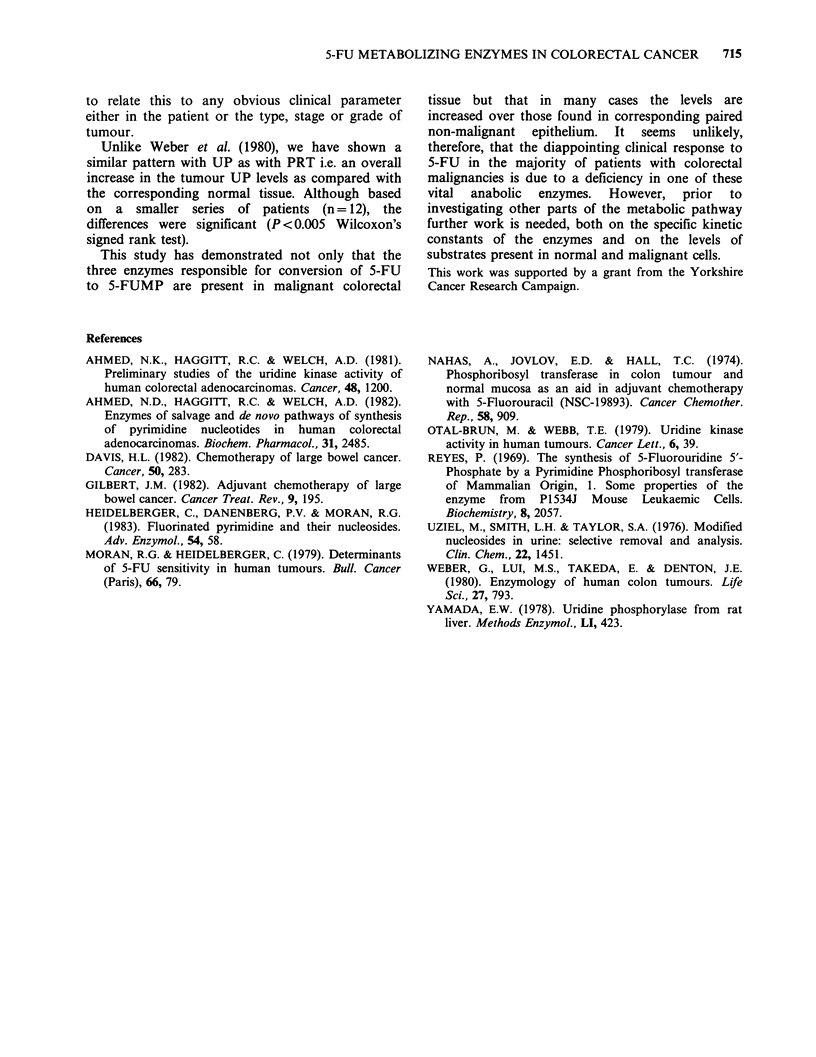# Comparative levels of tissue enzymes concerned in the early metabolism of 5-fluorouracil in normal and malignant human colorectal tissue.

**DOI:** 10.1038/bjc.1984.240

**Published:** 1984-11

**Authors:** P. J. Finan, P. A. Koklitis, E. M. Chisholm, G. R. Giles


*Br. J. Cancer* (1984), **50**, 711–715

### Short Communication

### Comparative levels of tissue enzymes concerned in the early metabolism of 5-fluorouracil in normal and malignant human colorectal tissue

P.J. Finan, P.A. Koklitis, E.M. Chisholm & G.R. Giles

*University Department of Surgery, St. James's University Hospital, Leeds, LS9 7TF, UK.*

Since its introduction nearly 30 years ago, 5-Fluorouracil (5-FU) has been used extensively both as a single agent and in combination therapy in the treatment of advanced colorectal malignancy (Gilbert, 1982 and Davis, 1982). This accumulation of experience has revealed a clinical response rate which remains stubbornly in the region of 20–25%. Clearly pre-selection of those patients that might benefit from treatment would be advantageous both in terms of an aggressive policy towards the likely "responders" and the avoidance of unnecessary chemotherapy to the majority of patients. Unfortunately, previous studies have not revealed clinical features that might predict the response to 5-FU leading some workers to investigate the biochemical characteristics of individual tumours; in particular the enzymes considered responsible for the metabolism of the drug (Moran & Heidelberger, 1979).

In its original form 5-FU is inactive and, in order to exert its cytotoxic effect, has to be converted to one of its active nucleotides down one of several metabolic pathways. In the first of these, long considered the most important 5-FU is converted to 5-fluorodeoxyuridine 5′-monophosphate (FdUMP). This nucleotide is a potent inhibitor of thymidylate synthetase and, by interfering with the supply of thymidine triphosphate, affects the synthesis of DNA. More recently it has been suggested that not all the cytotoxic action of 5-FU can be explained in this way and that a further important effect is incorporation of 5-FU into RNA (Heidelberger *et al.*, 1983). Such an action requires conversion of 5-FU to 5-fluorouridine 5′-monophosphate (FUMP) either directly using the enzyme phosphoribosyl transferase (PRT) or indirectly via 5-fluorouridine, using the enzymes uridine phosphorylase (UP) and uridine kinase (UK).

There is, as yet, little data on the levels of these three enzymes in human colorectal epithelium. For this reason and because of their obvious importance in the initial steps of metabolism of 5-FU this study was designed to measure the levels of enzymes in a series of matched normal and malignant specimens of human colorectal tissue.

Twenty-eight patients presenting with primary malignant lesions of the colorectum were studied (males = 17, females = 11 with a mean age of 67.3 range 51–82). Details of the site, stage and grade of individual tumours are contained in Table I.

**Table I** Clinical details of 28 colonic tumours studied

| | | | |
|---|---|---|---|
| A. *Site* | Right Colon | | = 7 |
| | Left Colon | | = 9 |
| | Rectum | | = 12 |
| B. *Stage* | Dukes' A | | = 0 |
| | B | | = 13 |
| | C | | = 10 |
| | Distant metaseses | | = 5 |
| C. *Grade* | Well | 12 | |
| | Moderate | 12 | |
| | Poor | 3 | |
| | Unclassified | 1 | |

At the time of surgery samples of both normal and malignant colonic epithelium were taken from the freshly-excised specimens, snap frozen in liquid nitrogen and stored at −70°C. Tissue extracts were obtained by homogenising the specimens in an equal volume of ice-cold 10 mM tris/HCl buffer (pH 7.5), containing 10 mM mercaptoethanol, centrifuging at 100,000 *g* for 30 min at 4°C and collecting the supernatant to store at −70°C. All reagents except where otherwise stated were of analytical grade from BDH Chemicals Ltd. (Poole).

The assay of phosphoribosyl transferase (PRT) (EC 2.4.2.9) was adapted from the radioisotopic method described by Reyes (1969). Sixty $\mu$l of extract (10 mM tris/HCl, pH 7.5) was incubated at 37°C for 30 min with 20 $\mu$l of 6.0 mM [6-$^3$H] 5-FU (Amersham), 20 $\mu$l of 400 mM tris/HCl (pH 9.8) and 40 $\mu$l of 15.0 mM 5-phosphoribosyl-1-pyrophosphate in 15 mM $MgCl_2$. Twenty $\mu$l aliquots were taken at 0, 10, 20 and 30 min and mixed with 500 $\mu$l 750 mM ammonium acetate (pH 9.0). The amount of

Correspondence: G.R. Giles.
Received 17 May 1984; accepted 16 July 1984

© The Macmillan Press Ltd., 1984

[6-$^3$H]FUMP formed was measured by applying the mixture to a boronate affinity gel prepared as described by Uziel et al. (1976). The gel was eluted with 250 mM ammonium acetate (pH 8.8) to remove unconverted [6-$^3$H] 5FU and then with 100 mM formic acid to remove the [6-$^3$H] FUMP. The latter was counted using a Phillips PW 4540 liquid scintillation analyser and the specific activity of the PRT expressed as nmol [6-$^3$H] 5FUMP produced $h^{-1} g^{-1}$ protein. The protein content of the supernatant was determined using the Biuret method.

The assay of uridine kinase (UK) – (E.C. 2.7.1.48) was based on the radio-isotopic method described by Ahmed et al. (1981). Forty μl of extract in 10 mM tris HCl, pH 7.5 (600–900 μg protein) was incubated for 40 min at 37°C with 40 μl of 50 mM [5,6-$^3$H] uridine (Amersham), 40 μl of 69.5 mM ATP, 40 μl of 62.5 mM $MgCl_2$ and 40 μl of M tris/HCl (pH 7.5) containing 50 mM β-mercaptoethanol. Twenty μl aliquots were taken at 2, 10, 20, 30 and 40 min and spotted on DE-81 discs (Whatman, Biochemical Ltd.). Unconverted [5,6-$^3$H] uridine was eluted from the discs with a continuous stream of water. [5, 6-$^3$H] uridine monophosphate (UMP) was eluted from the disc in 1 ml of 0.1 M HCl/0.5 M NaCl into scintillation vials and counted in a liquid scintillation analyser (Phillips PW 4540). The specific activity of UK was expressed as nmols of [5,6-$^3$H] UMP $h^{-1} g^{-1}$ protein in extract.

Uridine phosphorylase (UP) – (EC 2.4.2.3) measurements were based on the spectrophotometric method described by Yamada et al. (1978). Extract (50 μl) in 10 mM tris/HCl (pH 7.5) was incubated at 37°C for 15 min with 1.35 ml of 111 mM $Na_2HPO_4/KH_2PO_4$ and 0.1 ml of 50 mM uridine. The reaction was stopped by the addition of 75 μl of perchloric acid. Control tubes had the uridine added after the perchloric acid. After centrifugation at 15,000 g for 10 min and adjustment of the pH to 10 with 5 M NaOH, the absorbance of UV light at 290 mM was measured. Protein concentration of the supernatant was determined using the Biuret method and the activity of the enzyme was expressed in mmol of uracil $h^{-1} g^{-1}$ protein in extract.

The values for the three enzymes PRT, UP and UK in matched normal and malignant tissue are shown in Figure 1a–c respectively, with the median values and interquartile range in Table II.

There is a wide range of values for PRT in normal colonic tissue, however, it is evident that the corresponding values in the matched malignant tissue are elevated as compared with the normal epithelium. There appears to be no correlation between the level of the enzyme and the site, stage or grade of tumour as demonstrated in Figure 2a–c. The difference between values of PRT in normal and malignant tissue are highly significant using the Wilcoxon signed rank test for non-parametric data ($P < 0.005$).

**Table II** The median values and interquartile ranges of 3 enzyme levels in normal and tumour tissue

| | *PRT* (*nmol $h^{-1} g^{-1}$*) (*n* = *22*) | *UP* (*mmol $h^{-1} g^{-1}$*) (*n* = *12*) | *UK* (*nmol $h^{-1} g^{-1}$*) (*n* = *18*) |
|---|---|---|---|
| Normal | 9.84 | 12.98 | 2.00 |
| | (7.29–11.69) | (9.33–26.91) | (1.72–2.74) |
| Tumour | 19.02 | 30.53 | 3.32 |
| | (13.80–25.65) | (24.74–51.25) | (2.28–4.59) |

A similar pattern is observed for values of UP although this is based on a smaller sample size (n = 12). Again, with three exceptions, the values obtained for malignant tissue are significantly higher than for the corresponding normal tissue ($P < 0.005$). There appears to be no relationship between the level of enzyme and site, stage or grade of tumour.

Finally, the values for the enzyme uridine kinase (UK) are shown in Figure 1c. Although many tumours exhibit the pattern previously seen with PRT and UP there are several cases where the levels in normal and malignant tissue are either equivalent or even reduced in the tumour. The significance of this is not readily apparent when one studies the tumours in question.

Although there appeared to be a linear relationship between levels of PRT and UK ($r = 0.75$, $P < 0.001$) in 18 cases where both enzymes were assayed on the same tissue no other correlation could be found, either between PRT and UP or UP and UK.

Despite the controversy that continues to surround the mechanism of cytotoxicity of 5-FU, be it inhibition of DNA synthesis or a direct effect on the maturation of RNA (Heidelberger et al., 1983), it is clear that to have any effect 5-FU must be converted to one of two active nucleotides, FdUMP or FUMP. It is evident therefore that the enzymes responsible for these reactions should be studied in human material in an attempt to explain the relative resistance to 5-FU seen in 75–80% of colorectal malignancies.

Studies on cultured cells which have been selected for their resistance to 5-FU have shown decreased levels of the enzyme PRT. Similarly decreased levels of UK have been detected in cells which are resistant to fluorouridine (Heidelberger et al., 1983).

There are little data at the present time on these anabolic enzymes in human colorectal tissue. Weber (1980) studying 9 cases of primary colorectal carcinoma, showed an increase in PRT levels in malignant versus normal tissue. This increase was of

**Figure 1a–c** Values for the three enzymes (a) phosphoribosyl transferase, (b) uridine phosphorylase and (c) uridine kinase in matched normal and malignant tissue.

the same order of magnitude as this present series (i.e. ×2). Nahas *et al.*, 1974 studied ~100 individual matched pairs of tissue and, although suggesting that a higher ratio of tumour PRT to normal PRT favoured a positive clinical response, it was not possible to relate enzyme values in individual patients to clinical response. We similarly have found a wide range of values for PRT both in normal and malignant tissue. Although the levels are in general elevated in the latter we have no information on the sensitivity of these individual tumours to 5-FU treatment.

Previous studies have shown that levels of UK are also elevated in malignant colorectal tissue (Weber, 1982) although the actual level may vary enormously (Otal-Brun & Webb, 1979 and Ahmed *et al.*, 1982). It is interesting to note that in 7 of our cases the level of UK in tumour tissue was either equivalent or less than the corresponding normal tissue. As with the results for PRT we were unable

**Figure 2a–c** Enzyme levels in relation to (a) site of primary tumour; (b) tumour stage and (c) histological grading.

to relate this to any obvious clinical parameter either in the patient or the type, stage or grade of tumour.

Unlike Weber *et al.* (1980), we have shown a similar pattern with UP as with PRT i.e. an overall increase in the tumour UP levels as compared with the corresponding normal tissue. Although based on a smaller series of patients (n = 12), the differences were significant ($P < 0.005$ Wilcoxon's signed rank test).

This study has demonstrated not only that the three enzymes responsible for conversion of 5-FU to 5-FUMP are present in malignant colorectal tissue but that in many cases the levels are increased over those found in corresponding paired non-malignant epithelium. It seems unlikely, therefore, that the diappointing clinical response to 5-FU in the majority of patients with colorectal malignancies is due to a deficiency in one of these vital anabolic enzymes. However, prior to investigating other parts of the metabolic pathway further work is needed, both on the specific kinetic constants of the enzymes and on the levels of substrates present in normal and malignant cells.

This work was supported by a grant from the Yorkshire Cancer Research Campaign.

### References

AHMED, N.K., HAGGITT, R.C. & WELCH, A.D. (1981). Preliminary studies of the uridine kinase activity of human colorectal adenocarcinomas. *Cancer*, **48**, 1200.

AHMED, N.D., HAGGITT, R.C. & WELCH, A.D. (1982). Enzymes of salvage and *de novo* pathways of synthesis of pyrimidine nucleotides in human colorectal adenocarcinomas. *Biochem. Pharmacol.*, **31**, 2485.

DAVIS, H.L. (1982). Chemotherapy of large bowel cancer. *Cancer*, **50**, 283.

GILBERT, J.M. (1982). Adjuvant chemotherapy of large bowel cancer. *Cancer Treat. Rev.*, **9**, 195.

HEIDELBERGER, C., DANENBERG, P.V. & MORAN, R.G. (1983). Fluorinated pyrimidine and their nucleosides. *Adv. Enzymol.*, **54**, 58.

MORAN, R.G. & HEIDELBERGER, C. (1979). Determinants of 5-FU sensitivity in human tumours. *Bull. Cancer* (Paris), **66**, 79.

NAHAS, A., JOVLOV, E.D. & HALL, T.C. (1974). Phosphoribosyl transferase in colon tumour and normal mucosa as an aid in adjuvant chemotherapy with 5-Fluorouracil (NSC-19893). *Cancer Chemother. Rep.*, **58**, 909.

OTAL-BRUN, M. & WEBB, T.E. (1979). Uridine kinase activity in human tumours. *Cancer Lett.*, **6**, 39.

REYES, P. (1969). The synthesis of 5-Fluorouridine 5′-Phosphate by a Pyrimidine Phosphoribosyl transferase of Mammalian Origin, 1. Some properties of the enzyme from P1534J Mouse Leukaemic Cells. *Biochemistry*, **8**, 2057.

UZIEL, M., SMITH, L.H. & TAYLOR, S.A. (1976). Modified nucleosides in urine: selective removal and analysis. *Clin. Chem.*, **22**, 1451.

WEBER, G., LUI, M.S., TAKEDA, E. & DENTON, J.E. (1980). Enzymology of human colon tumours. *Life Sci.*, **27**, 793.

YAMADA, E.W. (1978). Uridine phosphorylase from rat liver. *Methods Enzymol.*, **LI**, 423.